# Association of socioeconomic factors on mental health in children and young people with intellectual developmental disabilities of genetic origin

**DOI:** 10.1002/jcv2.70180

**Published:** 2026-10-13

**Authors:** Irene O. Lee, Jeanne Wolstencroft, Harriet Housby, Marianne B. M. van den Bree, Samuel J. R. A. Chawner, Jeremy Hall, Michael J. Owen, IMAGINE ID Consortium, David H. Skuse

**Affiliations:** ^1^ Paediatric Mental Health Sciences Centre Great Ormond Street Institute of Child Health University College London London UK; ^2^ Department of Psychology College of Health Medicine and Life Sciences Brunel University of London London UK; ^3^ The Division of Psychology and Language Sciences Faculty of Brain Sciences University College London London UK; ^4^ Centre for Neuropsychiatric Genetics and Genomics Division of Psychological Medicine and Clinical Neurosciences Cardiff University Cardiff UK; ^5^ Division of Psychological Medicine and Clinical Neurosciences Neuroscience and Mental Health Innovation Institute Cardiff University Cardiff UK; ^6^ IMAGINE ID Consortium Great Britain London UK

**Keywords:** children and young people, emotional and behavioural adjustment, intellectual developmental disability, mental health, prevalence, socioeconomic status

## Abstract

**Background:**

Children and young people (CYP) with intellectual developmental disability (IDD) have a high prevalence of mental health and behavioural problems, which might be influenced by socioeconomical status as well as the severity of their disabilities. This is however an understudied topic, and the present study investigated the association between mental health and socioeconomic deprivation as well as severity/type of disability in CYP with IDD of genetic origin.

**Methods:**

Participants comprised a national cohort of 2778 participants with diagnosed genetic disorders associated with IDD, 10.1 ± 4.3 years of age (mean ± SD), 55.7% males. The cohort was linked to routinely collected UK school record data from the National Pupil Database. Socioeconomical status was assessed by free school meals (FSM) eligibility, the index of multiple deprivation (IMD), and parental educational level. IDD severity/type was reported by reviewing statements of primary special educational needs. Participant's mental health diagnoses were established by parental report using the Development and Well‐Being Assessment. Parents also completed the Strengths and Difficulties Questionnaire (SDQ).

**Results:**

Socioeconomic factors including lower parental education, greater deprivation (lower IMD decile) and FSM eligibility were associated with the higher prevalence of mental health disorders, as well as greater difficulties in emotional and behavioural adjustment (as measured by the SDQ). In contrast, IDD severity/type was not linked to diagnostic characteristics nor the prevalence of emotional and behavioural difficulties.

**Conclusion:**

This study revealed that CYP with IDD of genetic origin living in relatively deprived circumstances had a high risk of mental health disorders and poor emotional and behavioural adjustment, irrespective of the severity/type of their learning disabilities. Tackling deprivation (such as FSM as indicator for targeted additional support) and enhancing access to support services (e.g., psychotherapy) could be by implication key strategies to improve the mental health of this vulnerable population with IDD of genetic aetiology.

## Key Points

What's known?

- The authors assessed mental health and behavioural problems in a nationally representative cohort of 2778 children and young people (CYP) with diagnosed intellectual developmental disabilities (IDD) of genetic aetiology, using data from the National Pupil Database and IMAGINE‐ID studies.
- Socioeconomic disadvantage (lower IMD deciles, FSM eligibility, and lower parental education) was consistently associated with poorer mental health (more psychiatric diagnoses, higher SDQ scores).

What's new?

- Severity of learning disabilities was not associated with greater mental health difficulties.
- Socioeconomic factors play a major role in shaping mental health outcomes among CYP with IDD.

What's relevant?

- Improved understanding of the mechanisms through which socioeconomic disadvantage contributes to poor mental health in CYP with IDD is essential, both for informing policy and designing interventions tailored to this vulnerable group.

## INTRODUCTION

Intellectual developmental disability (IDD) is characterised by significantly impaired intellectual functioning and deficits in adaptive behaviours (Adlington et al., 2019; Ilyas et al., 2020; Tassé et al., 2016). It affects 2%–3% population worldwide (Daily et al., 2000) and is the most common developmental disorder (Ilyas et al., 2020). Children and young people (CYP) with IDD have additional needs in multiple areas of their life compared to the general population including mental health problems (Einfeld et al., 2011; Emerson & Hatton, 2007b; Hughes‐McCormack et al., 2017). IDD is more prevalent in families with lower household income, reliance on welfare, and poorer housing conditions (Durkin et al., 2007, 2010; Emerson, 2012; Victora et al., 2003) and in those circumstances is linked with poorer general and mental health and educational outcomes (Emerson & Hatton, 2007a; Fauth et al., 2017). IDD of genetic aetiology is associated with a particularly high risk of neuropsychiatric disorders (Chawner et al., 2019; Wolstencroft et al., 2022).

Mental health outcomes among CYP with IDD are not uniform; the severity of cognitive impairment and associated neurodevelopmental disorders has been found to shape both vulnerability and resilience (Emerson & Einfeld, 2010; Totsika et al., 2011). Previous research suggests those with greater intellectual disabilities have more behavioural and emotional problems (Einfeld et al., 2006; Flynn et al., 2017; Hove & Havik, 2010). Mental health problems are more likely to persist for people with severe or profound IDD, whereas that for those with mild IDD may decrease over time (Einfeld et al., 2006). Social deprivation could amplify emotional and behavioural problems particularly in individuals with severe or complex needs, because of cumulative family stress, higher service barriers, and lower access to specialist support (Emerson et al., 2006; Hastings et al., 2014). Specific disorders, such as anxiety, depression, oppositional defiant disorder (ODD), and conduct disorder are more common in socioeconomically disadvantaged children, reflecting a double burden of vulnerability (Collishaw et al., 2004; McManus et al., 2016). Crucially, while neurodevelopmental outcomes are heavily influenced by biological pathways in CYP with genetic conditions, research indicates that up to 80% of the variation in their cognitive and adaptive outcomes is still driven by non‐genetic, social‐environmental factors. When socioeconomic deprivation occurs alongside a genetic syndrome, external stress undermines the family’s capacity to invest in specialised care and therapeutic interventions. Poverty does not merely add an extra burden; it directly interacts with the biological vulnerabilities of a genetic condition, establishing a population‐specific socioeconomic gradient that broad disability frameworks fail to capture (McKinney et al., 2024). Yet few studies have examined the association of socioeconomic factors on mental health of CYP with IDD of genetic origin.

We studied a cohort of CYP with IDD that were associated with rare genetic disorders (Wolstencroft et al., 2022). Our objective was to test the hypothesis that the association between IDD of genetic aetiology and mental health disorders was influenced by the CYP family's socioeconomic status (SES) (including index of multiple deprivation [IMD], free school meals [FSM] eligibility and parental education level), as well as the severity or type of disability.

## METHODS

### Study participants

Two thousand seven hundred seventy‐eight CYP (55.7% male) aged between 4 and 25 years (mean ± SD, 10.1 ± 4.3) took part in the UK‐wide Intellectual Disability and Mental Health: Assessing the Genomic Impact on Neurodevelopment (IMAGINE‐ID) study. IMAGINE‐ID recruited a nationally representative cohort of CYP with IDD of genetic aetiology between 2014 and 2019 (Wolstencroft et al., 2022). All CYP had developmental delay and/or an intellectual disability diagnosis made by a clinical care team. Participation required a genetic diagnosis from an accredited diagnostic laboratory. Recruitment to the study was by referral from 23 UK regional genetics centres (76%), self‐referrals or patient support groups (23.5%), and the Experiences of People with Copy Number Variants (ECHO, 0.5%) study (see https://www.cardiff.ac.uk/mrc‐centre‐neuropsychiatric‐genetics‐genomics/research/themes/developmentalpsychiatry/echo‐study‐cnv‐research). Approximately 37% (820 out of 2198) of participants had inherited a pathogenic familial variant. 96.0% (*n* = 2666) participants were living with at least one biological parent at the time of the recruitment, 2.7% (*n* = 76) were with their legal guardians and 1.3% did not provide information. Details of parental education level were provided (1095 fathers and 1201 mothers).

The study was approved by the London Square Research Ethics Committee in the UK (20/LO/1271). Written or online consent was obtained from the parents/caregivers of children under the age of 16, and (where feasible) participating CYP themselves when over the age of 16. This included permission for linkage to educational and clinical records (hospital episode data). The participant demographic information is listed in Table 1.

**TABLE 1:** Participant demographic information.

Demographic category	Frequency (% of total count)
Sex	2778
Male	1547 (55.7%)
Female	1231 (44.3%)
Age (year)	
Mean ± SD	10.1 ± 4.3
Median	6.0
Range	4.0–25.1
IMD decile	
1 (most deprived)	305 (11.0%)
2	289 (10.4%)
3	272 (9.8%)
4	258 (9.3%)
5	282 (10.2%)
6	266 (9.6%)
7	253 (9.1%)
8	274 (9.9%)
9	276 (9.9%)
10 (least deprived)	303 (10.9%)
Eligible for FSM	
No	1799 (64.8%)
Yes	979 (35.3%)
Primary SEN type	
Profound and multiple LD	226 (8.1%)
Severe LD	625 (22.5%)
Moderate LD	437 (15.7%)
Specific LD	208 (7.5%)
Speech language communication needs	474 (17.1%)
Autism spectrum disorder	461 (16.6%)
Social emotion mental health^a^	93 (3.3%)
Visual, hearing and multi‐sensory impairment	29 (1.0%)
Physical disability	71 (2.6%)
Other difficulty/disability	105 (3.8%)
Missing data	49 (1.8%)
Parental education level—Father/mother	1195/1201
Incomplete compulsory education	113 (10.3%)/82 (6.8%)
GCSEs or O‐level	383 (35.0%)/372 (31.0%)
Vocational training	79 (7.2%)/98 (8.2%)
A‐levels or highers	157 (14.3%)/194 (16.2%)
University degree	229 (20.9%)/291 (24.2%)
Higher or postgraduate degree	134 (12.2%)/164 (13.7%)

Abbreviations: A‐level = advanced level qualifications, FSM = free school meals; GCSE = general certificate of secondary education qualification, IMD = index of multiple deprivation (IMD decile 1 indicates the most deprived 10% of neighbourhoods, decile 10 the 10% least deprived neighbourhoods), LD = learning difficulty, O‐level = ordinary Level qualification, SD = standard deviation.

^a^ Category known as ‘Behavioural Emotional Social Difficulty’ before 2014–2015.

### Data sources and data description

Primary caregivers of the participants were invited to complete online questionnaires regarding their child. We obtained detailed information about CYP's medical history and related diagnoses from 80% of the families taking part in IMAGINE‐ID (*n* = 2230). A formal psychiatric evaluation was obtained by parental report with the Development and Well‐Being Assessment (DAWBA) (Goodman et al., 2000). Diagnoses, using DSM‐5 criteria, were assigned on the basis of information obtained from this assessment by two independent experienced clinicians. DAWBA records were shared with independent raters who had used the same instrument to conduct the 2017 UK National Survey of Children's Mental Health. Inter‐rater reliability was checked with 147 randomly chosen participants and all kappa values exceeded 0.7 (Wolstencroft et al., 2022).

Two thousand four hundred one primary caregivers (86%) completed the Strengths and Difficulties Questionnaire (SDQ) (Goodman, 1997), which is suitable for rating emotional and behavioural difficulties among children and adolescents with intellectual and developmental disabilities (Murray et al., 2020). SDQ scores were categorised into four severity bands, derived from a general population UK survey (see Supporting Information S1: Appendix S1). These bands allocate 80% of randomly selected UK children and adolescents to a ‘Close to Average’ range regarded as ‘normal’, 10% in the ‘Slightly Raised’ range of problems are regarded as ‘borderline’. 10% score in the ‘High’ or ‘Very High’ range, indicative of problems that have potential clinical significance (Youth in Mind, 2016).

All participants had been educated in English mainstream or special educational state‐funded schools. Personal data from primary caregiver online surveys were linked to education histories derived from the National Pupil Database (NPD) which is managed by the UK Department for Education (DfE) following secure and standardised application procedures. The NPD educational dataset was ideal for the investigation of this study as the education‐related data is routinely collected in the UK state schools. Educational information was provided between the period from 2006 to 2021 and was available on all 2778 participants in this study. It included information on primary special education need (SEN) type and an index of socioeconomic impoverishment (whether the pupil was eligible for FSM). Pupils could be classified as belonging to a different ‘primary SEN type’ in consecutive academic years. For the purpose of this analysis, we assigned a single primary SEN type for each pupil; the one most frequently recorded in their education history (Lee et al., 2024). The primary SEN types included: Profound and multiple learning difficulty (LD), Severe LD, Moderate LD, Specific LD, Speech and language communication needs (SLCN), Social emotional mental health, Autism spectrum disorder, Hearing, Visual and Multiple sensory impairment and Physical disability; and the prevalences are listed in Table 1.

Postcodes of participating family homes allowed us to assign an IMD decile according to the UK Office for National Statistics (ONS) (UK Government, 2021). IMD decile 1 indicates the most deprived 10% of neighbourhood whereas decile 10 denotes the 10% least deprived neighbourhoods. Information on parental education level (for father: *n* = 1095 and mother: *n* = 1201) was provided by the primary caregivers who completed the online questionnaires for this study.

### Statistical analysis

Chi‐square (*X* ^2^) tests were used to compare proportions of participants with mental health diagnosis (dependent variable) in categories including IMD decile, eligibility for FSM, parental education level and primary SEN type. We used parametric statistics to identify the associations between SDQ scores (dependent variable) and the above independent variables which were regarded as nominal variables. Measures of effect size are reported. These include Cramer's *V* or Phi coefficient as measures of strength of association between two categorical variables associated with a positive Chi‐Square test. Cohen's *d* is a standard measure of effect size used in *t*‐tests. Eta‐squared (*ŋ* ^2^) is a measure of effect size used in ANOVA *F*‐tests. A *p*‐value <0.05 was adopted in all the analyses in this study as a cut‐off of statistical significance. Following the statistical disclosure guidelines from the ONS, NPD counts of fewer than ten cannot be published and therefore, exact numbers for certain groups are not presented and suppression of the related data were performed. All data analyses were performed in SPSS version 28 on the ONS Secure Research Service platform.

## RESULTS

### Measures of general wellbeing

Primary caregivers rated the general health status of their child on a Likert scale (see note in Supporting Information S1: Table S1). Higher proportions of CYP (66.5%–76.8%) living in the least deprived areas (IMD deciles 6–10) were reported their general health as at least ‘Good’ than those living in deciles 1–5 (55.8%–69.1%); significantly larger proportions of participants (44.2% in IMD decile 1) were rated with no better than ‘Fair’ general health in the most deprived decile than those in the least deprived area (24.2% in IMD decile 10) (*χ* ^2^ = 59.94, *p* = 0.007, *V* = 0.67, *N* = 2249). These health discrepancies were also found when the cohort was divided into those who were or were not eligible for FSM: higher percentage (40.1%) of CYP with FSM eligibility was reported their general health as no better than ‘Fair’ than those without (27.7%) (*χ* ^2^ = 41.02, *p* < 0.0001, *V* = 0.135, *N* = 2249). There was a decreasing trend (from 66% to 16%) of proportions of participants with FSM eligibility as the IMD deciles increased and vice versa (from 34% to 84%) for those without (*X* ^2^ = 339.24, *p* < 0.0001, *V* = 0.388, *N* = 2249, see Supporting Information S1: Figure S1).

### Emotional and behavioural problems

#### SDQ and IMD decile

The SDQ subscale scores of CYP living in the IMD deciles 1–5 (more deprived areas) were significantly higher than those from the higher IMD deciles (least deprived areas), indicating relatively more severe emotional symptoms, conduct problems, hyperactivity, peer relationship problems and total difficulties (*F* between 3.84 and 10.29; *η* ^2^ = 0.014–0.037, all *p*‐values < 0.0001, see Figure 1).

**FIGURE 1:**
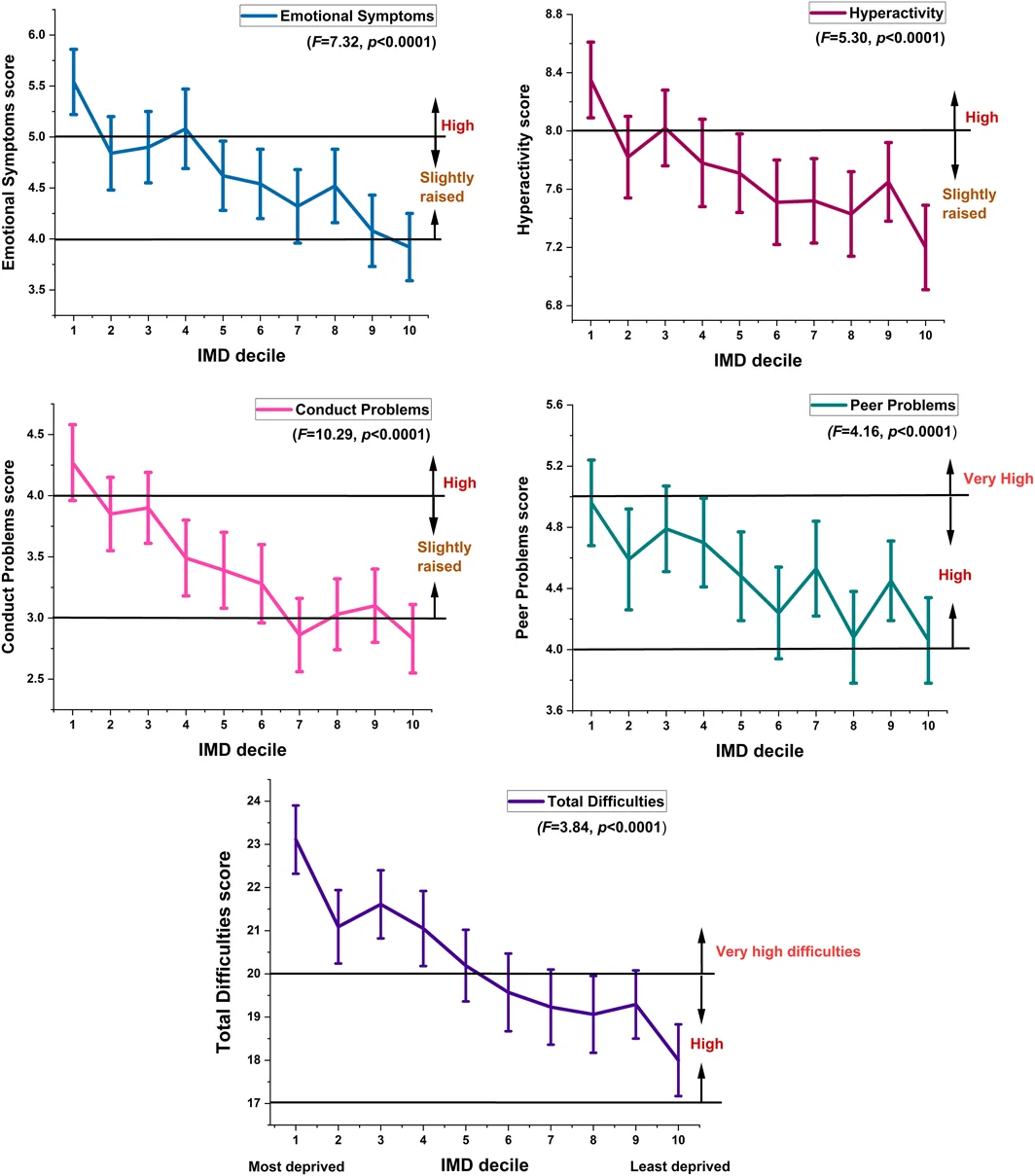
The SDQ subscale and total scores of the IMAGINE‐ID participants living in different IMD deciles. IMD decile 1 indicates the most deprived neighbourhood and IMD decile 10 denotes the least deprived neighbourhoods. Band classifications to show the degree of difficulties include ‘Slightly raised’, ‘High’ and ‘Very High’. IMD = index of multiple deprivation, SDQ = Strength and Difficulties Questionnaire.

#### SDQ and FSM eligibility

In this cohort, 35.3% participants were eligible for FSM compared to England's national figure (25.7%) in 2025 (UK‐Government, 2025). Participants who were eligible for FSM were rated to have significantly higher SDQ scores than those who were not eligible, including emotional symptoms, conduct problems, hyperactivity and peer relationship problems (*t* between −11.66 and −6.22; *d* = 0.243–0.48, all *p*‐values < 0.0001, see Table 2).

**TABLE 2:** The SDQ subscale and total scores of the cohort participants with and without eFSM.

SDQ score (mean ± 95%CI) [Band classification]	No eFSM	eFSM	*t*	*p*‐value
Emotional symptoms	4.3 ± 0.1 [Slightly raised]	5.2 ± 0.2 [High]	−7.72	<0.0001
Conduct problems	3.1 ± 0.1 [Slightly Raised]	4.1 ± 0.2 [High]	−9.66	<0.0001
Hyperactivity	7.5 ± 0.2 [Slightly Raised]	8.2 ± 0.1 [High]	−8.13	<0.0001
Peer relationship problems	4.3 ± 0.1 [High]	4.9 ± 0.2 [High]	−6.22	<0.0001
Prosocial behaviour	5.3 ± 0.1 [Very low]	5.1 ± 0.2 [Very low]	2.30	0.334
Total difficulties	19.1 ± 0.3 [High]	22.4 ± 0.5 [Very high]	−11.66	<0.0001

*Note*: Band classifications to show the degree of difficulties include ‘Slightly raised’, ‘High’ and ‘Very high’.

Abbreviations: CI = confidence interval, eFSM = free school meals eligibility, SDQ = Strengths and Difficulties Questionnaire.

#### SDQ and parental education level

We examined the relationship between parental educational achievements and CYP SDQ scores. Participants whose parents had lower education levels (such as incomplete compulsory education) obtained significantly higher SDQ subscale scores than those whose parents completed formal education. Problem areas included emotional symptoms, conduct problems, hyperactivity, peer relationships and total difficulties. The finding held for both fathers and mothers—fathers: *F* between 4.72 and 24.62, *η* ^2^ = 0.021–0.102, *N* = 1094, all *p*‐values < 0.001; mothers: *F* between 5.654 and 19.37, *η* ^2^ = 0.023–0.075, *N* = 1201, all *p*‐values < 0.001 (see Figure 2). Children of less educated parents were rated as having significantly lower prosocial behaviour scores (for fathers and mothers: *F* = 3.58, *p* = 0.003, *η* ^2^ = 0.016, *N* = 1094 and *F* = 3.25, *p* = 0.006, *η* ^2^ = 0.013, *N* = 1201 respectively).

**FIGURE 2:**
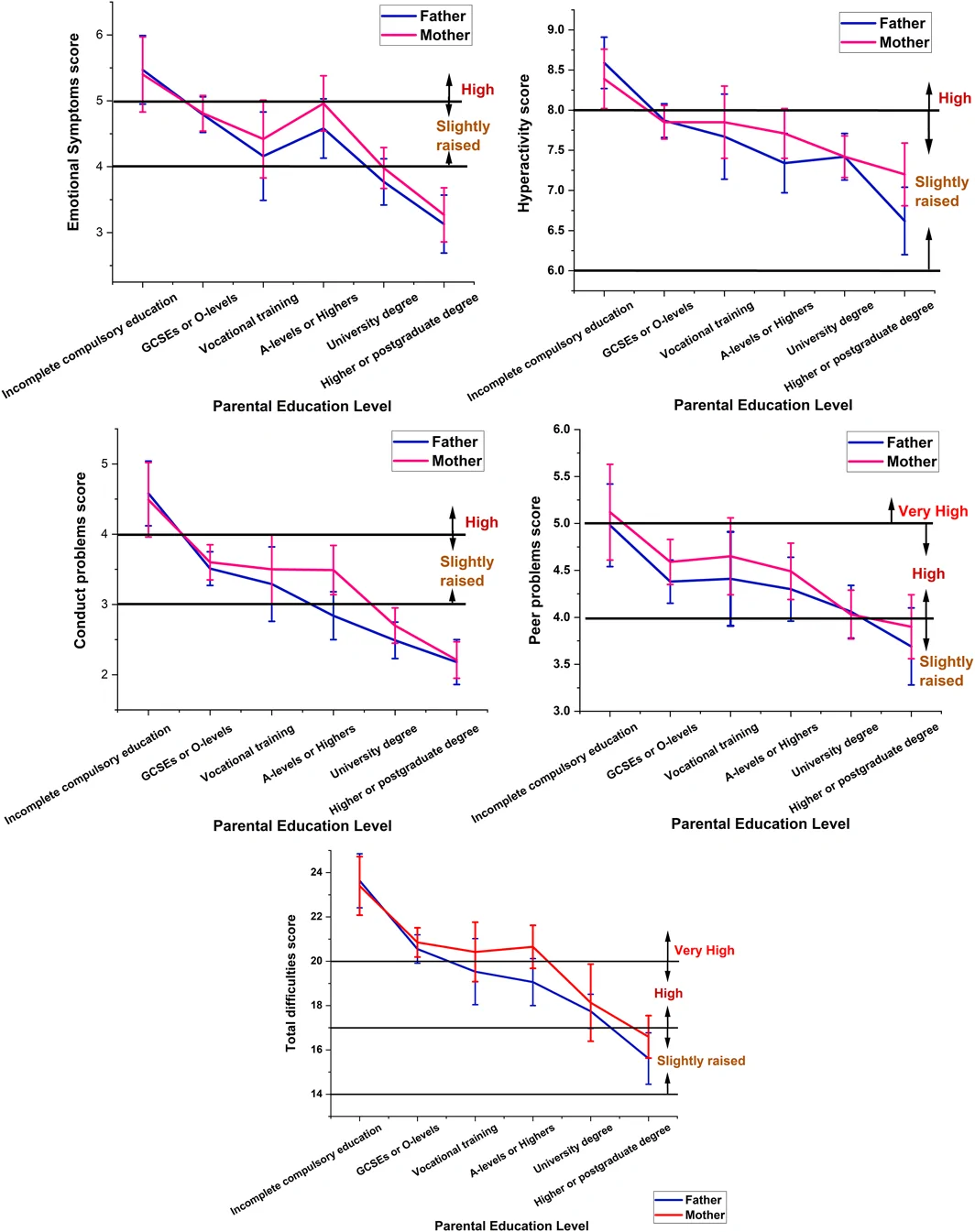
Comparisons of the SDQ subscale and total scores of participants for different levels of parental education. Band classifications to show the degree of difficulties include ‘Slightly raised’, ‘High’ and ‘Very High’. A‐level = advanced level qualifications, GCSE = general certificate of secondary education qualification, O‐level = ordinary level qualification, SDQ = Strength and Difficulties Questionnaire.

To test the independent effect of neighbourhood deprivation on child mental health, a multiple linear regression was conducted. Child age and sex were included in the model to adjust for fixed demographic characteristics. The overall regression model was statistically significant. Most importantly, after adjusting for child age and sex, IMD decile was a highly significant predictor of mental health difficulties (*β* = −0.200, *t* = −10.01, *p* < 0.001). This confirms that neighbourhood deprivation tracks mental health risks independently of basic child demographics.

#### Severity/type of IDD and SDQ score

We examined the association between the severity/type of IDD (indicated by primary SEN type) and emotional/behavioural adjustment. Both SDQ total and subscale scores differed between primary SEN types (*F* between 2.996 and 36.312; all *p*‐values < 0.001, *η* ^2^ = 0.011–0.123, *N* = 2329, see Figure 3 and Supporting Information S1: Figure S2). Those with profound and multiple learning difficulties (PMLD) had an average total SDQ score in the ‘slightly raised’ category relative to population norms. Those with severe learning difficulties (SLD) scored on average in the range of ‘high’ problem severity. Participants with multiple sensory impairments and those with physical disabilities (PD) obtained significantly lower scores than children with SLCN, social emotional mental health problems or autism spectrum disorders. Overall, CYP with more severe learning difficulties (PMLD, SLD) and those with physical disabilities were rated as having fewer emotion and behavioural difficulties than participants with less severe learning difficulties (see Figure 3 and Supporting Information S1: Table S2).

**FIGURE 3:**
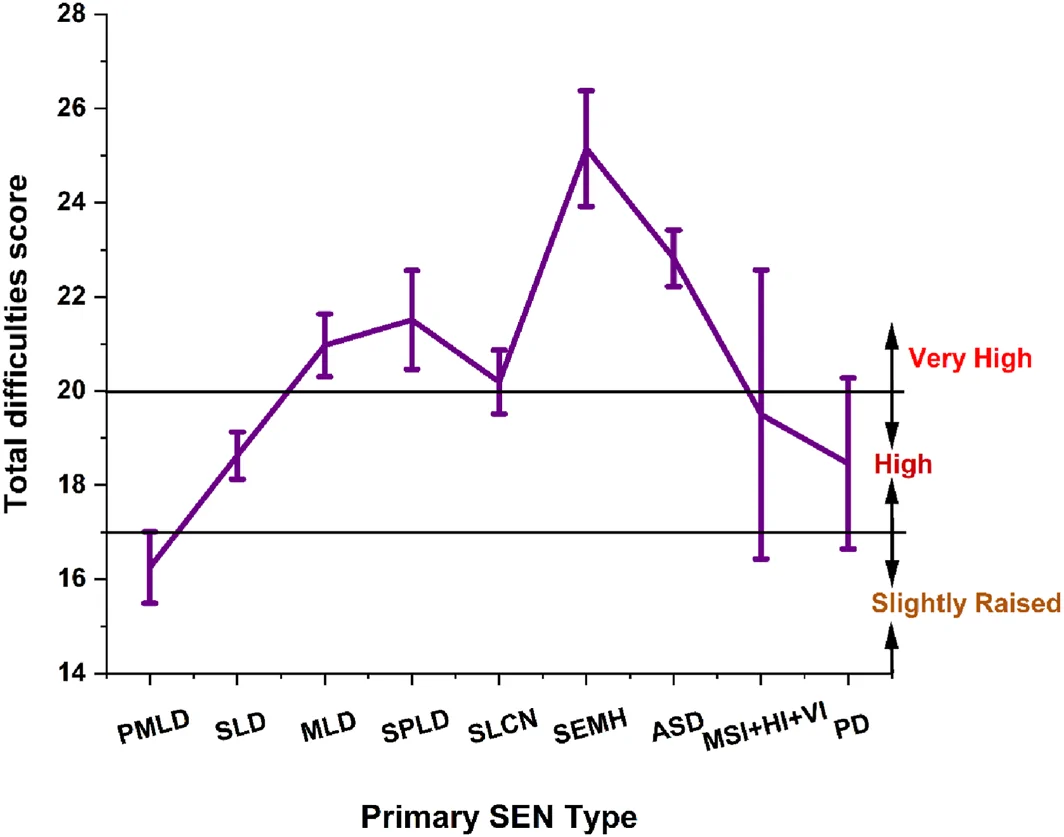
Comparison between total SDQ scores among the participants with different primary SEN types. Band classifications to show the degree of difficulties include ‘Slightly raised’, ‘High’ and ‘Very High’. ASD = autism spectrum disorder, MLD = moderate learning difficulty, MSI + HI + VI = combined multiple sensory impairment with hearing impairment and visual impairment, PD = physical disability, PMLD = profound and multiple learning difficulty, SDQ = Strength and Difficulties Questionnaire, SEMH = social emotional mental health, SEN = special education need, SLCN = speech and language communication needs, SLD = severe learning difficulty, SPLD = specific learning difficulty.

### Mental health diagnoses

DSM‐5 diagnoses were derived from the DAWBA. We found statistically significant differences in the proportion of CYP with mental health conditions between those living in the most and least deprived regions. There was a higher prevalence of mental health diagnoses in those living in more deprived regions (see Figure 4). Significant differences in prevalence were found in emotional disorders (*X* ^2^ = 10.17, *V* = 0.201, *p* = 0.038), general anxiety disorders (*X* ^2^ = 10.10, *V* = 0.254, *p* = 0.039), behavioural disorders (*X* ^2^ = 14.49, *V* = 0.225, *p* = 0.006), oppositional defiant disorders (*X* ^2^ = 14.10, *V* = 0.229, *p* = 0.007) and autism (*X* ^2^ = 15.34, *V* = 0.139, *p* = 0.004).

**FIGURE 4:**
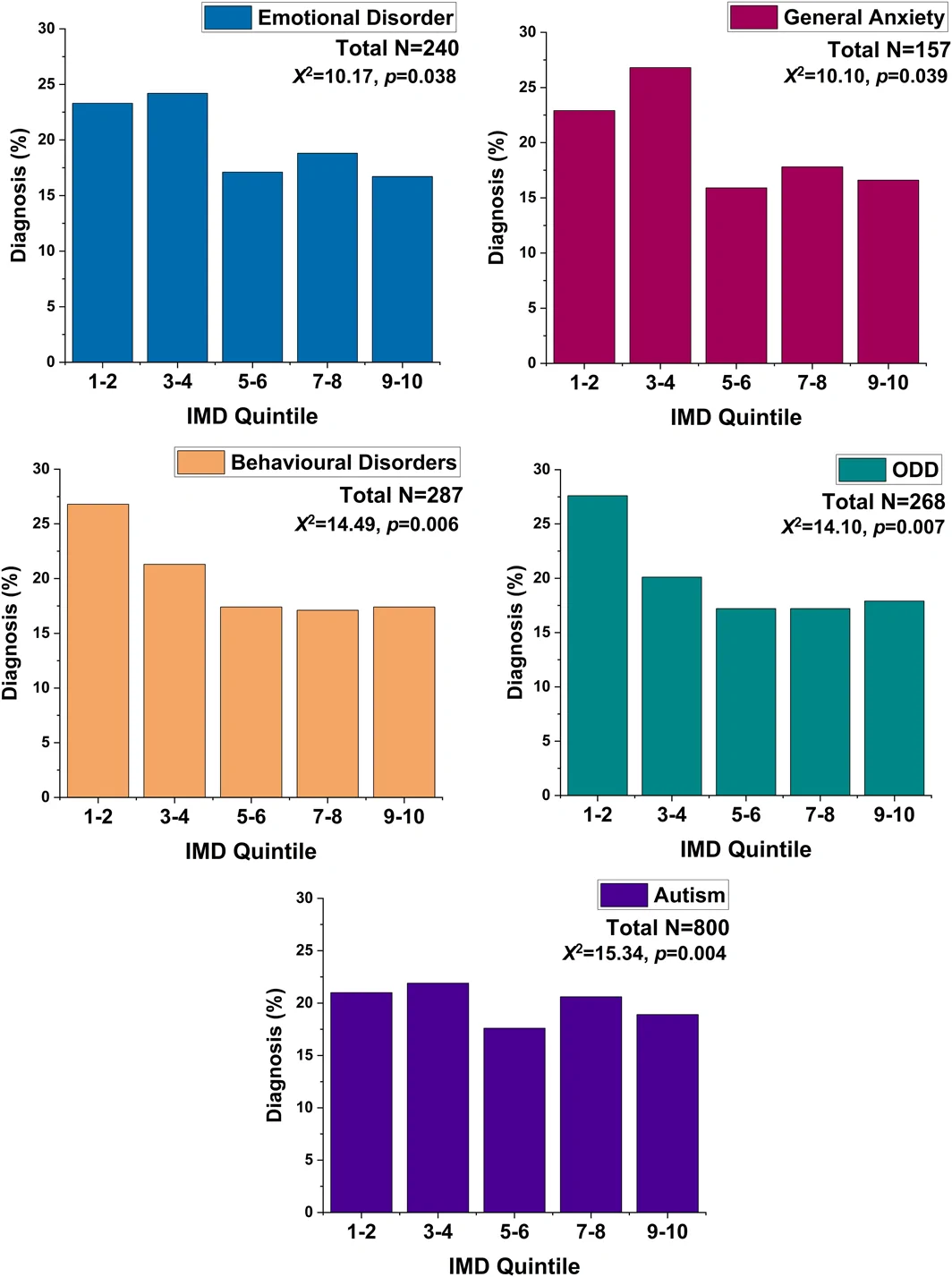
Prevalence of mental health diagnoses among participants, by IMD quintile. IMD = index of multiple deprivation, IMD quintile 1–2 = most deprived, IMD quintile 9–10 = least deprived, *N* = number of cases, ODD = oppositional defiant disorders.

#### Mental health diagnosis and FSM eligibility

Participants who were eligible for FSM had significantly higher relative risk of mental health diagnosis than other participants (all *p*‐values ranged <0.05–0.001, see Table 3), indicating higher prevalence of a wide range of mental health diagnoses.

**TABLE 3:** The relative risks of mental health diagnosis in those who were eligible for free school meals.

Mental health diagnosis	RR	95% CI	PHI
Emotional disorders	1.2	1.0–1.5^∗^	0.05
Separation anxiety	1.7	1.3–2.3^∗∗^	0.10
Major depression	2.5	1.9–3.3^∗∗^	0.19
ADHD	1.4	1.2–1.6^∗∗∗^	0.10
Autism	1.3	1.2–1.5^∗∗∗^	0.09
Behavioural disorders	1.6	1.4–1.9^∗∗∗^	0.13
ODD	1.6	1.4–1.8^∗∗∗^	0.13
Conduct disorder	2.3	1.9–2.8^∗∗∗^	0.20

Abbreviations: ADHD = attention deficit hyperactivity disorder, CI = confidence interval; ODD = oppositional defiant disorder, PHI = Phi coefficient, RRs = relative risks.

^∗^ *p* < 0.05.

^∗∗^ *p* < 0.01.

^∗∗∗^ *p* < 0.001.

#### Mental health diagnosis and parental education level

CYP of fathers with lower educational attainments were more likely to have mental health diagnoses, including hyperactivity (*X* ^2^ = 14.939, *p* = 0.011, *V* = 0.117, *N* = 1093, see Table 4), ADHD‐combined type (*X* ^2^ = 15.354, *p* = 0.009, *V* = 0.119, *N* = 1093), ODD (*X* ^2^ = 21.637, *p* < 0.001, *V* = 0.141, *N* = 1093), autism/pervasive developmental disorder (PDD) (*X* ^2^ = 26.179, *p* < 0.001, *V* = 0.155, *N* = 1093). This trend was less marked for mothers with lower education levels, but their CYP were reported to have a relatively higher prevalence of conduct disorder (*X* ^2^ = 17.952, *p* = 0.003, *V* = 0.122, *N* = 1197) and ODD (*X* ^2^ = 14.308, *p* = 0.014, *V* = 0.109, *N* = 1197).

**TABLE 4:** Prevalence of mental health diagnoses of participants by parental education levels.

Diagnosis	Father’s education level (*n* = 1093)				Mother’s education level (*n* = 1197)	
	Hyperactivity	ADHD‐combined	ODD	Autism/PDD	Conduct disorder	ODD
Incomplete compulsory education	31.0%	31.0%	22.1%	45.1%	6.2%	19.8%
GCSEs or O‐level	19.8%	19.1%	11.7%	38.6%	1.1%	11.1%
Vocational training	24.1%	22.8%	12.7%	44.3%	3.1%	16.5%
A‐levels or highers	16.0%	15.4%	9.0%	34.0%	2.1%	12.4%
University degree	18.4%	18.0%	8.3%	31.1%	<1.0%	9.3%
Higher or postgraduate degree	13.4%	13.4%	5.2%	19.4%	<1.0%	6.1%
*X* ^2^	14.939	15.354	21.637	26.179	17.952	14.308
Cramér's *V*	0.117	0.119	0.141	0.155	0.122	0.109
*p*‐values	0.011	0.009	<0.001	<0.001	0.003	0.014

*Note*: <1.0%: The counts are less than 10 and cannot be presented according to the statistical disclosure guidelines from the ONS. Only significant associations for each parent are listed here.

Abbreviations: ADHD = attention deficit hyperactivity disorder, A‐level = advanced level qualifications, GCSE = general certificate of secondary education qualification, ODD = oppositional defiant disorders, ONS = the Office for National Statistics, O‐level = ordinary level qualification, PDD = pervasive developmental disorder.

#### Mental health diagnosis and severity/type of IDD

The mental health diagnoses of participants with different primary SEN types varied without a clear pattern, including emotional disorder, general anxiety, hyperactivity, combined type of attention deficit hyperactivity disorder, ODD, conduct disorder and autism/PDD (*X* ^2^ between 23.44 and 256.534; *V* between 0.103 and 0.342, all *p*‐values < 0.003, *N* = 2193, see Table 5).

**TABLE 5:** Prevalence of mental health diagnosis of participants with different primary SEN type.

Diagnosis	Emotional disorder	General anxiety	Hyperactivity	ADHD combined	ODD	Conduct disorder	Autism
Primary SEN type							
Profound and multiple LD	7.2%	4.6%	7.2%	7.2%	3.9%	0.0%	19.6%
Severe LD	8.4%	5.3%	14.9%	14.7%	7.3%	0.9%	34.9%
Moderate LD	14.4%	10.4%	22.5%	22.5%	15.4%	1.5%	33.2%
Specific LD	9.4%	7.4%	26.8%	25.5%	12.1%	2.7%	27.5%
Speech language communication needs	7.5%	3.9%	22.2%	21.7%	9.6%	0.7%	28.4%
Social emotional mental health	18.7%	13.2%	47.3%	47.3%	33.0%	13.2%	40.7%
Autism spectrum disorder	14.7%	9.0%	28.4%	28.1%	17.3%	1.4%	78.1%
MSI + HI + VI	7.5%	7.5%	15.0%	15.0%	7.5%	0.0%	22.5%
Physical disability	11.7%	7.2%	14.4%	14.4%	9.0%	0.0%	23.4%
*X* ^2^	26.047	23.44	81.60	80.92	72.46	88.00	256.534
Cramér's *V*	0.109	0.103	0.193	0.192	0.187	0.206	0.342
*p*‐values	0.001	0.003	<0.001	<0.001	<0.001	<0.001	<0.001

Abbreviations: ADHD = attention deficit hyperactivity disorder, LD = learning difficulty, MSI + HI + VI = combined multiple sensory impairment with hearing impairment and visual impairment, ODD = oppositional defiant disorders, PDD = pervasive developmental disorder, SEN = special educational need.

## DISCUSSION

Our findings support the hypothesis that there is a substantial association between social deprivation and the emergence of mental health problems in CYP with IDD of genetic origin. Participants living in relatively disadvantaged neighbourhoods, who were eligible for FSM, and whose parents had lower levels of education, had more severe emotional and behavioural difficulties. They were also more likely to have a wide range of mental health diagnoses, including ODD, conduct disorder, emotional disorder, anxiety and autism. Our findings are consistent with previous reports that children with IDD face disproportionately high rates of mental health problems (Emerson & Hatton, 2007b; Hope et al., 2014; Patalay & Fitzsimons, 2016), and that these can be exacerbated by poverty and its associated stressors (Emerson, 2012; Emerson & Hatton, 2007a; Hulbert‐Williams et al., 2014; Totsika et al., 2011). In contrast to earlier research reports (Cooper et al., 2007; Eaton et al., 2021; Flynn et al., 2017), we did not find an association between greater severity (SEN type) of IDD and mental health difficulties. Instead, there was clear evidence of a link to socioeconomic disadvantage implying that social factors play an important role in shaping mental health outcomes.

We do not currently have evidence to indicate the mechanisms that underly these associations. It is important to emphasise that all the data we have about the participant's behaviour comes from parental report. However, in the open text DAWBA section provided by caregivers, there were usually detailed anecdotal descriptions of family life. For many vulnerable families economic hardship and increasing parental stress is precipitated by the need for at least one parent to be a full‐time carer for their disabled child. Families living in socioeconomically deprived areas have relatively limited access to services that provide early intervention, therapy, and respite (Emerson & Hatton, 2007b).

In a significant proportion of cases (about one third) participants recruited into the IMAGINE‐ID cohort had inherited a pathogenic familial variant. We have previously reported that CYP with inherited variants tended to live in socioeconomically more deprived regions than those with de novo mutations (Wolstencroft et al., 2022). From the information we have obtained on parental education, we know that many carriers themselves are likely to have experienced learning difficulties. Parents of children with intellectual disabilities who have relatively low levels of education and income face greater barriers to health and social care access, partly due to reduced awareness of entitlements and service pathways (Emerson, 2012).

Several previous studies have reported relatively high levels of psychopathology among children with severe or complex disabilities (Cooper et al., 2007; Dekker & Koot, 2003; Einfeld et al., 2006), but the aetiology of those mental health difficulties remains unclear. Our results show that the primary SEN type, as determined by education authorities, is not strongly related to mental health outcomes, as reported by parents. Our findings imply, in contrast, that environmental disadvantage may be a more powerful determinant of mental health than the intrinsic characteristics of the disability. While disability‐related characteristics undoubtedly influence developmental trajectories, socioeconomic factors exert a stronger and more consistent influence on mental health outcomes than the severity or classification of the child's SEN types.

Accordingly, our findings imply that if social care was to target familial deprivation, through parent training and community support, the adverse mental health impact of socioeconomic hardship on a proportion of CYP with IDD could be mitigated (Totsika et al., 2011). Our findings reinforce the importance of integrating social determinants of health frameworks into disability policy, and have implications for the associated work of child mental health services (Marmot et al., 2020). We recommend that future studies should explicitly include measures of family functioning, parental mental health, and service access; these are potential mediating or moderating factors in determining outcomes of CYP's social and behavioural adjustment. Prospective longitudinal research is necessary to identify mechanisms of change; we acknowledge the current study measures associations in cross‐sectional data. To that end, we have already undertaken a longitudinal study, following up this cohort over 5 years: these data are currently undergoing analysis. We hope to identify causative mechanisms relevant to longer‐term outcomes, as the cohort's median age moves from middle childhood into adolescence.

We recognise limitations to the interpretation of our findings. Measures of participant's mental health and adaptive functioning in this study were provided by parents/caregivers. We do not have independent assessments from schools or colleges for the majority of participants in IMAGINE‐ID. However, teacher report measures were collected in a sub‐group of the IMAGINE‐ID cohort assessed in person, and findings can be read in previously published work (Chawner et al., 2019). There was good consistency between teacher and parent reports in this subgroup. Although the study recruited nearly 3000 families, not all completed our standardised measures (e.g., SDQ, DAWBA). Information about parental mental health and the families' living conditions was limited, and the measure of socioeconomic deprivation was inferred from postcode data. There could be potential bias in the completion of our standardised assessments by families where the caregivers had their own cognitive difficulties, although we offered personal support to those who struggled to complete our assessments online. Our cohort was representative of the general population: we have published data showing that the distribution of participant families in relation to ethnicity and IMD deciles is close to national figures (Wolstencroft et al., 2022). We acknowledge that our measure of IDD severity was based on a single (though representative) measure of each child's primary special educational need. In practice, pupils were classified as having a range of different primary SEN types during the course of their education in successive academic years.

Another limitation of the current study relates to a single measure of SES (IMD score); future research would benefit from moving beyond proxy indicators towards more robust, direct measures of socioeconomic position, such as occupational status, material hardship, and community‐level deprivation. A key limitation of this study is its cross‐sectional design, which precludes any definitive conclusions regarding causal pathways. While our findings demonstrated a strong link between socioeconomic factors and mental health difficulties in CYP with IDD, the directional nature of this relationship remains unclear. It is possible that reverse causation is at play. For instance, families supporting a child with IDD and co‐occurring mental health challenges will face significant caregiving demands. These demands can severely restrict parental employment opportunities, increase healthcare and specialised care costs, and ultimately cause families to drift into socioeconomic disadvantage over time. Although empirical evidence for this ‘social drift’ hypothesis in the context of childhood disability remains limited compared to the evidence for social causation, it represents a plausible mechanism that future longitudinal research must disentangle. Future research utilising comparative datasets includes control group of CYP without IDD to rigorously test whether the observed socio‐economic associations are unique to IDD or are consistent across broader childhood disability classifications.

## CONCLUSIONS

This study demonstrated a clear association between the prevalence of mental health difficulties and the social environment in which CYP with IDD are living. Families who reside in deprived areas and socioeconomically disadvantaged are reported by their caregivers to have significantly higher levels of emotional and behavioural difficulties than CYP from more advantaged backgrounds, irrespective of their genetic condition. Contrary to some previous studies of children with IDD, we did not find any significant relationship between measures of disability severity and emotional or behavioural adjustment. Overall, findings from the IMAGINE‐ID cohort imply that mental health inequalities among CYP with IDD are driven primarily by socioeconomic disadvantage rather than disability severity. Tackling deprivation and enhancing access to support services, such as mental health and social care, are therefore key strategies to improve mental health equity for this vulnerable population.

## AUTHOR CONTRIBUTIONS

**Irene O. Lee**: Resources; formal analysis; writing—original draft preparation; writing—review and editing. **Jeanne Wolstencroft**: Data curation; writing—review and editing. **Harriet Housby**: Data curation; writing—review and editing. **Marianne B. M. van den Bree**: Conceptualization; methodology; writing—review and editing. **Samuel J. R. A. Chawner**: Conceptualization; methodology; data curation; writing—review and editing. **Jeremy Hall**: Conceptualization; methodology; writing—review and editing. **Michael J. Owen**: Conceptualization; methodology; writing—review and editing. **IMAGINE ID Consortium**: Conceptualization; methodology; writing—review and editing. **David H. Skuse**: Conceptualization; methodology; writing—review and editing.

## CONFLICT OF INTEREST STATEMENT

The authors declare no conflicts of interest.

## ETHICAL CONSIDERATIONS

The study was approved by the London Square Research Ethics Committee in the UK. The approval date of the last amendment was 28 January 2022 (20/LO/1271). The informed consent was obtained from all subjects and/or their legal guardian(s) in a written or online format.

## Supporting information

Supporting Information S1

## Data Availability

The data from the Department for Education (DfE), UK that support the findings in this paper are not available for data sharing according to the guidelines of the DfE and the Office for National Statistics (ONS). Other IMAGINE ID research data from this study are available from the authors upon application (see details of the application of data access in https://imagine‐id.org/research‐data‐access/; contact person: Corresponding Author). This work contains statistical data from ONS which is Crown Copyright. The use of the ONS statistical data in this work does not imply the endorsement of the ONS in relation to the interpretation or analysis of the statistical data. This work uses research datasets which may not exactly reproduce National Statistics aggregates.

## References

[jcv270180-bib-0001] Adlington, K. , Smith, J. , Crabtree, J. , Win, S. , Rennie, J. , Khodatars, K. , Rosser, E. , & Hall, I. (2019). Improving access to genetic testing for adults with intellectual disability: A literature review and lessons from a quality improvement project in East London. American Journal of Medical Genetics Part B: Neuropsychiatric Genetics, 180(8), 566–575. 10.1002/ajmg.b.32732 31077569

[jcv270180-bib-0002] Chawner, S. , Owen, M. J. , Holmans, P. , Raymond, F. L. , Skuse, D. , Hall, J. , & van den Bree, M. B. M. (2019). Genotype‐phenotype associations in children with copy number variants associated with high neuropsychiatric risk in the UK (IMAGINE‐ID): A case‐control cohort study. Lancet Psychiatry, 6(6), 493–505. 10.1016/S2215-0366(19)30123-3 31056457

[jcv270180-bib-0003] Collishaw, S. , Maughan, B. , Goodman, R. , & Pickles, A. (2004). Time trends in adolescent mental health. Journal of Child Psychology and Psychiatry, 45(8), 1350–1362. 10.1111/j.1469-7610.2004.00335.x 15482496

[jcv270180-bib-0004] Cooper, S. A. , Smiley, E. , Finlayson, J. , Jackson, A. , Allan, L. , Williamson, A. , Mantry, D. , & Morrison, J. (2007). The prevalence, incidence, and factors predictive of mental ill‐health in adults with profound intellectual disabilities. Journal of Applied Research in Intellectual Disabilities, 20(6), 493–501. 10.1111/j.1468-3148.2007.00401.x

[jcv270180-bib-0005] Daily, D. K. , Ardinger, H. H. , & Holmes, G. E. (2000). Identification and evaluation of mental retardation. American Family Physician, 61(4), 1059–1067, Article no. 1070. https://www.ncbi.nlm.nih.gov/pubmed/10706158 10706158

[jcv270180-bib-0006] Dekker, M. C. , & Koot, H. M. (2003). DSM‐IV disorders in children with borderline to moderate intellectual disability. I: Prevalence and impact. Journal of the American Academy of Child & Adolescent Psychiatry, 42(8), 915–922. 10.1097/01.CHI.0000046892.27264.1A 12874493

[jcv270180-bib-0007] Durkin, M. , Schupf, N. , Stein, Z. , & Susser, M. (2007). Childhood cognitive disability. In Wallace R. B. (Ed.), Public health and preventive medicine (15th ed., pp. 1173–1184). McGraw‐Hill.

[jcv270180-bib-0008] Durkin, M. S. , Maenner, M. J. , Meaney, F. J. , Levy, S. E. , DiGuiseppi, C. , Nicholas, J. S. , Kirby, R. S. , Pinto‐Martin, J. A. , & Schieve, L. A. (2010). Socioeconomic inequality in the prevalence of autism spectrum disorder: Evidence from a U.S. cross‐sectional study. PLoS One, 5(7), e11551. 10.1371/journal.pone.0011551 PMC2902521 20634960

[jcv270180-bib-0009] Eaton, C. , Tarver, J. , Shirazi, A. , Pearson, E. , Walker, L. , Bird, M. , Oliver, C. , & Waite, J. (2021). A systematic review of the behaviours associated with depression in people with severe‐profound intellectual disability. Journal of Intellectual Disability Research, 65(3), 211–229. 10.1111/jir.12807 33426741

[jcv270180-bib-0010] Einfeld, S. L. , Ellis, L. A. , & Emerson, E. (2011). Comorbidity if intellectual disability and mental disorder in children and adolescents: A systematic review. Journal of Intellectual and Developmental Disability, 36(2), 137–143. 10.1080/13668250.2011.572548 21609299

[jcv270180-bib-0011] Einfeld, S. L. , Piccinin, A. M. , Mackinnon, A. , Hofer, S. M. , Taffe, J. , Gray, K. M. , Bontempo, D. E. , Hoffman, L. R. , Parmenter, T. , & Tonge, B. J. (2006). Psychopathology in young people with intellectual disability. JAMA, 296(16), 1981–1989. 10.1001/jama.296.16.1981 PMC2422867 17062861

[jcv270180-bib-0012] Emerson, E. (2012). Deprivation, ethnicity and the prevalence of intellectual and developmental disabilities. Journal of Epidemiology and Community Health (1979‐), 66(3), 218–224. 10.1136/jech.2010.111773 20889590

[jcv270180-bib-0013] Emerson, E. , & Einfeld, S. (2010). Emotional and behavioural difficulties in young children with and without developmental delay: A bi‐national perspective. Journal of Child Psychology and Psychiatry, 51(5), 583–593. 10.1111/j.1469-7610.2009.02179.x 20015191

[jcv270180-bib-0014] Emerson, E. , Graham, H. , & Hatton, C. (2006). The measurement of poverty and socio‐economic position in research involving people with intellectual disabilities. In International review of research in mental retardation (pp. 77–108). Academic Press.

[jcv270180-bib-0015] Emerson, E. , & Hatton, C. (2007a). Contribution of socioeconomic position to health inequalities of British children and adolescents with intellectual disabilities. American Journal on Mental Retardation, 112(2), 140–150. 10.1352/0895-8017(2007)112[140:COSPTH]2.0.CO;2 17295554

[jcv270180-bib-0016] Emerson, E. , & Hatton, C. (2007b). Mental health of children and adolescents with intellectual disabilities in Britain. British Journal of Psychiatry, 191(6), 493–499. 10.1192/bjp.bp.107.038729 18055952

[jcv270180-bib-0017] Fauth, R. , Thompson, M. , & Penny, A. (2017). Neighbourhood deprivation and young children’s wellbeing: Evidence from the Millennium Cohort Study. Longitudinal and Life Course Studies, 8(3), 295–316.

[jcv270180-bib-0018] Flynn, S. , Vereenooghe, L. , Hastings, R. P. , Adams, D. , Cooper, S. A. , Gore, N. , Hatton, C. , Hood, K. , Jahoda, A. , Langdon, P. E. , McNamara, R. , Oliver, C. , Roy, A. , Totsika, V. , & Waite, J. (2017). Measurement tools for mental health problems and mental well‐being in people with severe or profound intellectual disabilities: A systematic review. Clinical Psychology Review, 57, 32–44. 10.1016/j.cpr.2017.08.006 28821007

[jcv270180-bib-0019] Goodman, R. (1997). The Strengths and Difficulties Questionnaire: A research note. Journal of Child Psychology and Psychiatry, 38(5), 581–586. https://acamh.onlinelibrary.wiley.com/doi/epdf/10.1111/j.1469‐7610.1997.tb01545.x 10.1111/j.1469-7610.1997.tb01545.x 9255702

[jcv270180-bib-0020] Goodman, R. , Ford, T. , Richards, H. , Gatward, R. , & Meltzer, H. (2000). The development and well‐being assessment: Description and initial validation of an integrated assessment of child and adolescent psychopathology. Journal of Child Psychology and Psychiatry, 41(5), 645–655. 10.1111/j.1469-7610.2000.tb02345.x 10946756

[jcv270180-bib-0021] Hastings, R. P. , Beck, A. , & Hill, C. (2014). The impact of life stressors on the mental health of families of children with intellectual disabilities. Journal of Applied Research in Intellectual Disabilities, 27(5), 393–404. 10.38106/LMRJ.2022.4.1-03

[jcv270180-bib-0022] Hope, S. , Pearce, A. , Whitehead, M. , & Law, C. (2014). Family employment and child socioemotional behaviour: Longitudinal findings from the UK Millennium Cohort Study. Journal of Epidemiology & Community Health, 68(10), 950–957. 10.1136/jech-2013-203673 PMC4174125 24889054

[jcv270180-bib-0023] Hove, O. , & Havik, O. E. (2010). Developmental level and other factors associated with symptoms of mental disorders and problem behaviour in adults with intellectual disabilities living in the community. Social Psychiatry and Psychiatric Epidemiology, 45(1), 105–113. 10.1007/s00127-009-0046-0 19347237

[jcv270180-bib-0024] Hughes‐McCormack, L. A. , Rydzewska, E. , Henderson, A. , MacIntyre, C. , Rintoul, J. , & Cooper, S. A. (2017). Prevalence of mental health conditions and relationship with general health in a whole‐country population of people with intellectual disabilities compared with the general population. BJPsych Open, 3(5), 243–248. 10.1192/bjpo.bp.117.005462 PMC5620469 29034100

[jcv270180-bib-0025] Hulbert‐Williams, L. , Hastings, R. , Owen, D. M. , Burns, L. , Day, J. , Mulligan, J. , & Noone, S. J. (2014). Exposure to life events as a risk factor for psychological problems in adults with intellectual disabilities: A longitudinal design. Journal of Intellectual Disability Research, 58(1), 48–60. 10.1111/jir.12050 23627774

[jcv270180-bib-0026] Ilyas, M. , Mir, A. , Efthymiou, S. , & Houlden, H. (2020). The genetics of intellectual disability: Advancing technology and gene editing. F1000Research, 9, 22. 10.12688/f1000research.16315.1 PMC6966773 31984132

[jcv270180-bib-0027] Lee, I. O. , Wolstencroft, J. , Housby, H. , van den Bree, M. B. M. , Chawner, S. J. R. A. , Hall, J. , Consortium, I. I. , & Skuse, D. H. (2024). The inequity of education, health and care plan provision for children and young people with intellectual and developmental disabilities. Journal of Intellectual Disability Research, 68(10), 1167–1183. 10.1111/jir.13139 PMC11753480 38777582

[jcv270180-bib-0028] Marmot, M. , Allen, J. , Boyce, T. , Goldblatt, P. , & Morrison, J. (2020). Health equity in England: The Marmot review 10 years on. https://www.instituteofhealthequity.org/resources‐reports/marmot‐review‐10‐years‐on/the‐marmot‐review‐10‐years‐on‐full‐report.pdf 10.1136/bmj.m693 32094110

[jcv270180-bib-0029] McKinney, W. S. , Williford, D. N. , Abbeduto, L. , & Schmitt, L. M. (2024). The impact of social‐environmental factors on IQ in syndromic intellectual developmental disabilities. Journal of Clinical and Translational Science, 8(1), e59. 10.1017/cts.2024.510 PMC11036438 38655457

[jcv270180-bib-0030] McManus, S. , Bebbington, P. , & Brugha, T. (2016). Adult psychiatric morbidity survey: Survey of mental health and wellbeing, England, 2014. NHS England. Retrieved from https://digital.nhs.uk/data‐and‐information/publications/statistical/adult‐psychiatric‐morbidity‐survey/adult‐psychiatric‐morbidity‐survey‐survey‐of‐mental‐health‐and‐wellbeing‐england‐2014

[jcv270180-bib-0031] Murray, C. A. , Hastings, R. P. , & Totsika, V. (2020). Clinical utility of the parentreported Strengths and Difficulties Questionnaire as a screen for emotional and behavioural difficulties in children and adolescents with intellectual disability. British Journal of Psychiatry, 218(6), 323–325. 10.1192/bjp.2020.224 33198827

[jcv270180-bib-0032] Patalay, P. , & Fitzsimons, E. (2016). Correlates of mental illness and wellbeing in children: Are they the same? Results from the UK millennium cohort study. Journal of the American Academy of Child & Adolescent Psychiatry, 55(9), 771–783. 10.1016/j.jaac.2016.05.019 27566118

[jcv270180-bib-0033] Tassé, M. J. , Luckasson, R. , & Schalock, R. L. (2016). The relation between intellectual functioning and adaptive behavior in the diagnosis of intellectual disability. Intellectual and Developmental Disabilities, 54(6), 381–390. 10.1352/1934-9556-54.6.381 27893317

[jcv270180-bib-0034] Totsika, V. , Hastings, R. P. , Emerson, E. , Lancaster, G. A. , & Berridge, D. M. (2011). A population‐based investigation of behavioural and emotional problems and maternal mental health: Associations with autism spectrum disorder and intellectual disability. Journal of Child Psychology and Psychiatry, 52(1), 91–99. 10.1111/j.1469-7610.2010.02295.x 20649912

[jcv270180-bib-0035] UK Government . (2021). Populations by Index of multiple deprivation (IMD) decile, England and Wales, 2020. Retrieved from https://www.ons.gov.uk/peoplepopulationandcommunity/populationandmigration/populationestimates/adhocs/13773populationsbyindexofmultipledeprivationimddecileenglandandwales2020

[jcv270180-bib-0036] UK‐Government . (2025). Schools, pupils and their characteristics, academic year 2024/25. Retrieved from https://explore‐education‐statistics.service.gov.uk/find‐statistics/school‐pupils‐and‐their‐characteristics/2024‐25

[jcv270180-bib-0037] Victora, C. G. , Wagstaff, A. , Schellenberg, J. A. , Gwatkin, D. , Claeson, M. , & Habicht, J. P. (2003). Applying an equity lens to child health and mortality: More of the same is not enough. Lancet, 362(9379), 233–241. 10.1016/S0140-6736(03)13917-7 12885488

[jcv270180-bib-0038] Wolstencroft, J. , Wicks, F. , Srinivasan, R. , Wynn, S. , Ford, T. , Baker, K. , Chawner, S. , Hall, J. , van den Bree, M. B. M. , Owen, M. J. , Study, I. , Skuse, D. , Raymond, F. L. , Erwood, M. , Lafont, A. , Timur, H. , Ye, Z. , Walker, S. , … & Printzlau, F. (2022). Neuropsychiatric risk in children with intellectual disability of genetic origin: IMAGINE, a UK national cohort study. Lancet Psychiatry, 9(9), 715–724. 10.1016/S2215-0366(22)00207-3 PMC9636306 35932790

[jcv270180-bib-0039] Youth in Mind . (2016). Scoring the strengths and Difficulties Questionnaire for ages 4–17. In Scoring the SDQ. Youth in Mind. Retrieved from https://www.sdqinfo.org/py/sdqinfo/c0.py

