# Supplementary material for: Association of socioeconomic factors on mental health in children and young people with intellectual developmental disabilities of genetic origin
Source: JCPP Adv. 2026 Oct 13:e70180. Online ahead of print. doi: 10.1002/jcv2.70180 (PMC13661137; doi:10.1002/jcv2.70180)
Supplement: Supplementary file 1 [file JCV2-9999-e70180-s001.docx]

**Association of socioeconomic factors on mental health in children and young people with intellectual developmental disabilities of genetic origin**

**Supporting Information**

**Table of Contents**

[**Appendix S1.** More information about the SDQ measures.	2](#_Toc237868527)

[**Table S1.** Comparisons of general health conditions of the participants with free school meals eligibility and living in different index of multiple deprivation (IMD) deciles.	4](#_Toc237868528)

[**Figure S1.** Distribution of participants with free school meals eligibility living in different index of multiple deprivation (IMD) deciles.	5](#_Toc237868529)

[**Figure S2.** Comparisons of Strength and Difficulties Questionnaire (SDQ) subscale scores of those with different primary special education need (SEN) types.	6](#_Toc237868530)

[**Table S2.** Post Hoc test of the total and subscales of Strength and Difficulties questionnaire (SDQ) scores between each primary special education need (SEN) types.	7](#_Toc237868531)

[**References**	10](#_Toc237868532)

# **Appendix S1.** More information about the SDQ measures.

The Strengths and Difficulties Questionnaire (SDQ)[1] is a brief behavioural screening questionnaire about 2–17 year olds, and is a globally recognised instrument for assessing the mental health status for children and young people [2]. SDQ scoring provides an overview to evaluate children and young people’s emotional and behavioural adjustment in dimensional terms [3, 4].

There are 25 items in the SDQ which comprises of 5 scales in 5 items each that measure: emotional symptoms; conduct problems; hyperactivity, impulsivity, and inattention difficulties; peer relationship problems; and prosocial behaviour. The first four of these scales are combined to make a total difficulties score. Higher scores are indicative of a greater mental health difficulty and scores above 90th percentile indicate a high probability of a diagnosable psychiatric disorder; whereas a lower score on the prosocial behaviour implies greater difficulties [4, 5]. The SDQ has been used as a standardised assessment to evaluate emotional and behavioural difficulties of children and adolescents with intellectual and developmental disabilities [3].

The SDQ scores were categorised into four bands based on a general population UK survey: 80% of UK children score in a “Close to Average” range regarded as ‘normal’, 10% score “Slightly Raised” range as ‘borderline’, and 10% score “High” or “Very High” range regarded as ‘abnormal’ [5, 6].

Categorisation bands for SDQ scores are listed in the following table to identify the correct classification for each score.

For Parent/Carer completed SDQ:

| **Classification for each SDQ subscale score** | **Close to**  **Average**  (80% pop) | **Slightly raised**  **(/lowered)**  (10% pop) | **High**  **(/Low)**  (5% pop | **Very high**  **(/very low)**  (5% pop) |
| --- | --- | --- | --- | --- |
| Emotional problems score | 0-3 | 4 | 5-6 | 7-10 |
| Conduct problems score | 0-2 | 3 | 4-5 | 6-10 |
| Hyperactivity score | 0-5 | 6-7 | 8 | 9-10 |
| Peer problems score | 0-2 | 3 | 4 | 5-10 |
| Prosocial score | 8-10 | 7 | 6 | 0-5 |
| **Total difficulties score** | 0-13 | 14-16 | 17-19 | 20-40 |

Note: pop=population.

For further information regarding the scoring process, please visit: <https://sdqscore.org/>.

**Table S1.** Comparisons of general health conditions of the participants with free school meals eligibility and living in different index of multiple deprivation (IMD) deciles.

|  | Very Good | Good | Fair | Bad | Very Bad | *χ*2 | *p*-value |
| --- | --- | --- | --- | --- | --- | --- | --- |
| General Health * |  |  |  |  |  | 41.02 | <0.0001 |
| No eFSM | 29.3% | 43.0% | 22.4% | 4.2% | 1.1% |  |  |
| eFSM | 21.8% | 38.1% | 30.3% | 7.9% | 1.9% |  |  |
| IMD decile |  |  |  |  |  | 50.46 | 0.004 |
| 1 (Most deprived) | 21.5% | 34.3% | 31.8% | 12.4% | |  |  |
| 2 | 22.0% | 42.6% | 28.7% | 6.7% | |  |  |
| 3 | 24.0% | 40.3% | 30.3% | 5.4% | |  |  |
| 4 | 26.9% | 40.6% | 24.1% | 8.5% | |  |  |
| 5 | 30.0% | 39.2% | 24.7% | 6.2% | |  |  |
| 6 | 26.3% | 40.0% | 26.3% | 7.3% | |  |  |
| 7 | 29.2% | 46.8% | 19.0% | 5.1% | |  |  |
| 8 | 26.9% | 40.5% | 26.0% | 6.6% | |  |  |
| 9 | 26.8% | 46.1% | 21.5% | 5.7% | |  |  |
| 10 (Least deprived) | 33.9% | 43.2% | 18.7% | 4.3% | |  |  |

Note: * The primary caregivers rated the child’s general health for a question asking, “*How is his/her health in general*? on a Likert scale with choices of “Very Good”, “Good”, “Fair”, “Bad” and “Very Bad”.

Percentages highlighted in purple: The counts of the category "Very Bad" were all less than 10 which cannot be shown according to the ONS guidelines, and therefore the categories “Bad” and “Very Bad” were combined.

**Figure S1.** Distribution of participants with free school meals eligibility living in different index of multiple deprivation (IMD) deciles.

**Figure S2.** Comparisons of Strength and Difficulties Questionnaire (SDQ) subscale scores of those with different primary special education need (SEN) types.

Note: PMLD=Profound and multiple learning difficulty; SLD=Severe learning difficulty; MLD=Moderate Learning difficulty; SPLD=Specific learning difficulty; SLCN=Speech and language communication needs; SEMH=Social emotional mental health; ASD=Autism spectrum disorder; MSI+HI+VI=Combined multiple sensory impairment with hearing impairment and visual impairment; PD=Physical disability.

**Table S2.** Post Hoc test of the total and subscales of Strength and Difficulties questionnaire (SDQ) scores between each primary special education need (SEN) types.

Note: only shown *p*<0.05. SE=Standard error; PMLD=Profound and multiple learning difficulty; SLD=Severe learning difficulty; MLD=Moderate Learning difficulty; SPLD=Specific learning difficulty; SLCN=Speech and language communication needs; SEMH=Social emotional mental health; ASD=Autism spectrum disorder; MSI+HI+VI=Combined multiple sensory impairment with hearing impairment and visual impairment; PD=Physical disability.

| **Primary SEN Type comparisons** | | **Mean difference** | **SE** | **p-values** |
| --- | --- | --- | --- | --- |
| **Total Difficulties score** | |  |  |  |
| **PMLD** | **SLD** | -2.377 | 0.536 | <0.001 |
|  | **MLD** | -4.725 | 0.567 | <0.001 |
|  | **SPLD** | -5.26 | 0.669 | <0.001 |
|  | **SLCN** | -3.939 | 0.56 | <0.001 |
|  | **SEMH** | -8.899 | 0.848 | <0.001 |
|  | **ASD** | -6.569 | 0.56 | <0.001 |
| **SLD** | **MLD** | -2.348 | 0.429 | <0.001 |
|  | **SPLD** | -2.883 | 0.556 | <0.001 |
|  | **SLCN** | -1.563 | 0.42 | 0.009 |
|  | **SEMH** | -6.523 | 0.763 | <0.001 |
|  | **ASD** | -4.193 | 0.42 | <0.001 |
| **MLD** | **SEMH** | -4.175 | 0.784 | <0.001 |
|  | **ASD** | -1.844 | 0.458 | 0.003 |
| **SPLD** | **SEMH** | -3.64 | 0.861 | 0.001 |
| **SLCN** | **SEMH** | -4.96 | 0.78 | <0.001 |
|  | **ASD** | -2.63 | 0.45 | <0.001 |
| **SEMH** | **MSI** | 5.648 | 1.444 | 0.004 |
|  | **PD** | 6.688 | 1.076 | <0.001 |
| **ASD** | **PD** | 4.357 | 0.867 | <0.001 |
| **Emotional Symptoms** | |  |  |  |
| **PMLD** | **SLD** | -0.921 | 0.227 | 0.002 |
|  | **MLD** | -2.105 | 0.24 | <0.001 |
|  | **SPLD** | -2.419 | 0.283 | <0.001 |
|  | **SLCN** | -1.777 | 0.237 | <0.001 |
|  | **SEMH** | -2.708 | 0.359 | <0.001 |
|  | **ASD** | -2.465 | 0.237 | <0.001 |
| **SLD** | **MLD** | -1.184 | 0.181 | <0.001 |
|  | **SPLD** | -1.498 | 0.235 | <0.001 |
|  | **SLCN** | -0.856 | 0.178 | <0.001 |
|  | **SEMH** | -1.787 | 0.323 | <0.001 |
|  | **ASD** | -1.544 | 0.178 | <0.001 |
| **SPLD** | **PD** | 1.488 | 0.398 | 0.009 |
| **SLCN** | **ASD** | -0.688 | 0.19 | 0.014 |
| **SEMH** | **PD** | 1.778 | 0.455 | 0.004 |
| **ASD** | **PD** | 1.535 | 0.357 | 0.001 |
| **Conduct Problems** | |  |  |  |
| **PMLD** | **MLD** | -1.413 | 0.203 | <0.001 |
|  | **SPLD** | -1.392 | 0.239 | <0.001 |
|  | **SLCN** | -1.385 | 0.2 | <0.001 |
|  | **SEMH** | -3.618 | 0.303 | <0.001 |
|  | **ASD** | -1.682 | 0.2 | <0.001 |
| **SLD** | **MLD** | -0.787 | 0.153 | <0.001 |
|  | **SLCN** | -0.759 | 0.15 | <0.001 |
|  | **SEMH** | -2.992 | 0.273 | <0.001 |
|  | **ASD** | -1.057 | 0.15 | <0.001 |
| **MLD** | **SEMH** | -2.205 | 0.28 | <0.001 |
| **SPLD** | **SEMH** | -2.226 | 0.308 | <0.001 |
| **SLCN** | **SEMH** | -2.233 | 0.279 | <0.001 |
| **SEMH** | **ASD** | 1.936 | 0.279 | <0.001 |
|  | **MSI** | 2.776 | 0.516 | <0.001 |
|  | **PD** | 2.656 | 0.385 | <0.001 |
| **Hyperactivity** | **No significant differences in post hoc tests.** | | | |
| **Peer Problems** | |  |  |  |
| **PMLD** | **SLD** | -0.641 | 0.187 | 0.028 |
|  | **MLD** | -0.974 | 0.198 | <0.001 |
|  | **SPLD** | -1.111 | 0.233 | <0.001 |
|  | **SLCN** | -0.67 | 0.195 | 0.028 |
|  | **SEMH** | -1.654 | 0.296 | <0.001 |
|  | **ASD** | -1.848 | 0.195 | <0.001 |
| **SLD** | **SEMH** | -1.013 | 0.266 | 0.006 |
|  | **ASD** | -1.207 | 0.146 | <0.001 |
| **MLD** | **ASD** | -0.874 | 0.16 | <0.001 |
| **SPLD** | **ASD** | -0.737 | 0.202 | 0.012 |
| **SLCN** | **SEMH** | -0.985 | 0.272 | 0.013 |
|  | **ASD** | -1.179 | 0.157 | <0.001 |
| **ASD** | **PD** | 1.27 | 0.302 | 0.001 |
| **Prosocial Behaviour** | |  |  |  |
| **PMLD** | **SLD** | -1.336 | 0.222 | <0.001 |
|  | **MLD** | -3.064 | 0.235 | <0.001 |
|  | **SPLD** | -2.859 | 0.277 | <0.001 |
|  | **SLCN** | -2.806 | 0.232 | <0.001 |
|  | **SEMH** | -1.576 | 0.352 | <0.001 |
|  | **ASD** | -1.139 | 0.232 | <0.001 |
|  | **MSI** | -3.646 | 0.555 | <0.001 |
|  | **PD** | -2.301 | 0.385 | <0.001 |
| **SLD** | **MLD** | -1.728 | 0.178 | <0.001 |
|  | **SPLD** | -1.523 | 0.231 | <0.001 |
|  | **SLCN** | -1.471 | 0.174 | <0.001 |
|  | **MSI** | -2.311 | 0.533 | <0.001 |
| **MLD** | **SEMH** | 1.488 | 0.325 | <0.001 |
|  | **ASD** | 1.926 | 0.19 | <0.001 |
| **SPLD** | **SEMH** | 1.282 | 0.357 | 0.015 |
|  | **ASD** | 1.72 | 0.24 | <0.001 |
| **SLCN** | **SEMH** | 1.23 | 0.323 | 0.007 |
|  | **ASD** | 1.668 | 0.187 | <0.001 |
| **SEMH** | **MSI** | -2.07 | 0.599 | 0.025 |
| **ASD** | **MSI** | -2.508 | 0.537 | <0.001 |

**References**

1.	Goodman, R. The strengths and difficulties questionnaire: A research note. *J Child Psychol Psychiatry*. **38**, 581–86 (1997). DOI: <https://acamh.onlinelibrary.wiley.com/doi/epdf/10.1111/j.1469-7610.1997.tb01545.x>.

2.	Terapia. Scoring strengths and difficulties questionnaire for age 4-17 or 18+ 2020 [Available from: <https://terapia.co.uk/wp-content/uploads/2020/05/SDQ-scoring_Instructions_4-18-years.pdf>.

3.	Murray, C. A., Hastings R. P., Totsika V. Clinical utility of the parentreported strengths and difficulties questionnaire as a screen for emotional and behavioural difficulties in children and adolescents with intellectual disability. *Br J Psychiatry*. **218**, 323-25 (2020). DOI: 10.1192/bjp.2020.224.

4.	Goodman, A., Goodman R. Strengths and difficulties questionnaire as a dimensional measure of child mental health. *Journal of the American Academy of Child and Adolescent Psychiatry*. **48** (4), 400-3 (2009). DOI: 10.1097/CHI.0b013e3181985068.

5.	Goodman, R. Psychometric properties of the strengths and difficulties questionnaire. *J Am Acad Child Adolesc Psychiatry*. **40**, 1337-45 (2001). DOI: 10.1097/00004583-200111000-00015.

6.	Youth in Mind. Scoring the strengths and difficulties questionnaire for ages 4–17. In scoring the sdq: Youth in Mind; 2016 [Available from: <https://www.sdqinfo.org/py/sdqinfo/c0.py>.
